# Supplementary material for: In Vivo Biodistribution of Respirable Solid Lipid Nanoparticles Surface-Decorated with a Mannose-Based Surfactant: A Promising Tool for Pulmonary Tuberculosis Treatment?
Source: Nanomaterials (Basel). 2020 Mar 21;10(3):568. doi: 10.3390/nano10030568 (PMC7153707; doi:10.3390/nano10030568)
Supplement: Supplementary file 1 [file nanomaterials-10-00568-s001.pdf]

Supplementary materials

In Vivo Biodistribution of Respirable Solid Lipid Nanoparticles Surface-Decorated with a Mannose-Based Surfactant: A Promising Tool for Pulmonary Tuberculosis Treatment?

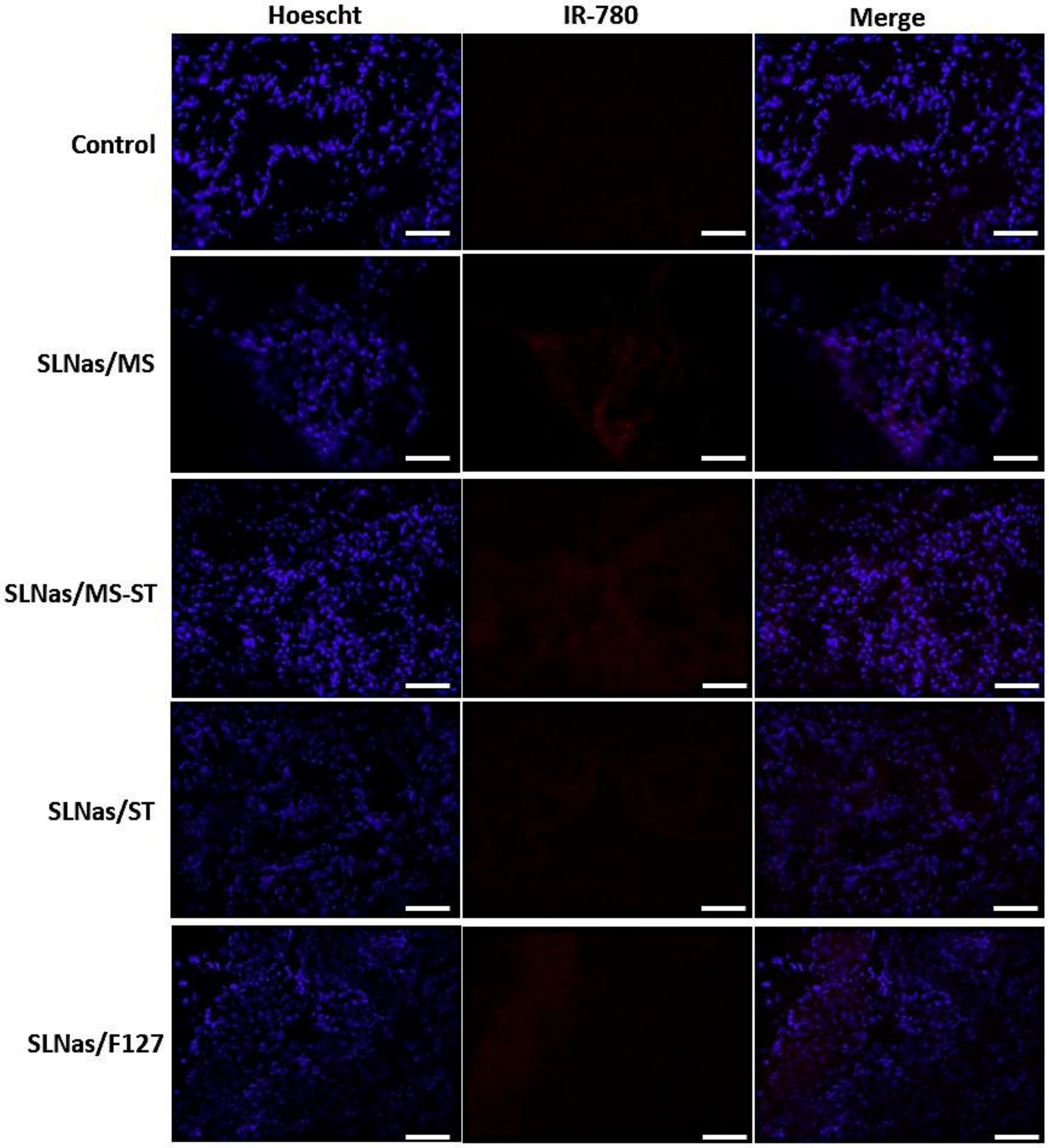

Figure 1. Representative fluorescence microscopy images of lung dissections at 24 h post-exposure. Pictures were taken with a 20x magnification. From left to right, first frame: images taken using the filter for Hoechst label (cells), second frame: images taken using the filter for IR-780 label (SLNas), third frame: merge, showing lungs cells in blue and SLNas in red. Scale bars = 100 μm.
